# Fullerenol C_60_(OH)_36_: Antioxidant, Cytoprotective, Anti-Influenza Virus Activity, and Self-Assembly in Aqueous Solutions and Cell Culture Media

**DOI:** 10.3390/antiox13121525

**Published:** 2024-12-13

**Authors:** Alina A. Borisenkova, Mikhail Y. Eropkin, Nadezhda I. Konovalova, Anna V. Titova, Maria A. Markova, Zhanna B. Lyutova, Anton S. Mazur, Victor P. Sedov, Vera A. Orlova, Anna N. Lykholay, Diana N. Orlova, Alexandr V. Arutyunyan

**Affiliations:** 1 Radiation Technology Department, St. Petersburg State Institute of Technology, Technical University, 190013 St. Petersburg, Russia; 2 Petersburg Nuclear Physics Institute Named by B.P. Konstantinov of National Research Centre “Kurchatov Institute”, 188300 Gatchina, Russia; 3 Smorodintsev Research Institute of Influenza, 197022 St. Petersburg, Russia; 4 Research Resource Center “Magnetic Resonance Research Methods”, St. Petersburg State University, 199034 St. Petersburg, Russia; 5 Khlopin Radium Institute, 194021 St. Petersburg, Russia; 6 Research Resource Center “Molecular and Cell Technologies”, St. Petersburg State University, 199034 St. Petersburg, Russia

**Keywords:** fullerenol, physicochemical characterization, reactive oxygen species, antioxidant scavenging activity, self-assembly, biocompatibility, toxicity, cytoprotection, anti-influenza activity

## Abstract

Viral infections and many other dangerous diseases are accompanied by the development of oxidative stress, which is a consequence of an increase in the level of the reactive oxygen species (ROS). In this regard, the search for effective antioxidants remains highly relevant. We tested fullerenol C_60_(OH)_36_ in the context of the connection between its self-assembly in aqueous solutions and cell culture media, antiradical activity, UV cytoprotective action, and antiviral activity against international reference strains of influenza virus A(H1N1)pdm09, A(H3N2), and B subtypes in vitro on the MDCK cell line. Various characterization techniques, including Fourier-transform infrared spectroscopy (FTIR), Raman spectroscopy, NMR and ESR spectrometry, MALDI-TOF mass spectrometry, thermal analysis (TGA and DSC), dynamic light-scattering (DLS), and ζ-potential measurements, were used to confirm the production of fullerenol and study its self-assembly in aqueous solutions and cell culture media. Fullerenol C_60_(OH)_36_ demonstrated the ability to scavenge ^•^DPPH, ^•^OH, O_2_^•−^ radicals and ^1^O_2_ and was non-toxic in the range of the studied concentrations (up to 200 μg/mL) when incubated with MDCK cells for 24 h. In addition, fullerenol exhibited a cytoprotective effect under UV irradiation (EC_50_ = 29.7 ± 1.0 μM) and showed moderate activity against human influenza viruses of subtypes A(H1N1)pdm09 (SI = 9.9 ± 4.6) and A(H3N2) (SI = 12.5 ± 1.3) when determined by the hemagglutination assay (HA-test) and the MTT assay. At the same time, C_60_(OH)_36_ was ineffective in vitro against the actual strain of influenza B virus (Victoria lineage). The high bioavailability of fullerenol in combination with its cytoprotective effect, as well as its antiradical and antiviral activity combined with a relatively low toxicity, allows to consider it a promising compound for biomedical applications.

## 1. Introduction

In the regulation of vital processes, an important role belongs to the reactive oxygen species (ROS), which, in low concentrations, are normal participants in many typical cellular mechanisms, despite their reactivity and potential toxicity [1]. At the same time, excessive formation of ROS is one of the leading mechanisms in the pathogenesis of many diseases [2,3]. For example, viral infections are usually accompanied by inflammation and a decrease in the effectiveness of endogenous antioxidant defense mechanisms, which ultimately contributes to the development of oxidative stress resulting from increased levels of ROS [4,5]. In this regard, it is pathogenetically justified to prevent the production, neutralize, and eliminate excess oxygen radicals and free radical oxidation products [5].

It is well known that fullerenes are free radical scavengers. The functionalization of fullerenes, which makes them soluble in water, can significantly increase their bioavailability. One of the common methods for functionalizing fullerenes is the synthesis of their hydroxyl derivatives, namely, fullerenols, by various methods [6]. Fullerenols C_60_(OH)_n_ with different hydroxyl groups numbers (2 ≤ *n* ≤ 44) were obtained by changing the conditions of the synthesis reactions. This, along with such beneficial properties as antioxidant [7,8,9,10,11,12] activity, high lipophilicity [13], and cytoprotective properties under UV- [14,15] and gamma-irradiation [16,17], makes fullerenols very promising for biomedical applications. Thus, fullerene derivatives could be used as antiviral drugs with nonspecific action [18]. Due to their powerful antioxidant activity, fullerenols could be effective in neurodegenerative diseases associated with oxidative stress [19,20].

The antiviral activity of fullerene derivatives depends on their structure, as noted by the authors in [18]. In addition, water-soluble fullerene derivatives of different structures vary in their ability to scavenge free radicals. This observation is associated with the differences in electron affinities due to the functionalization of the fullerene surface with different chemical groups [11,21], and with such properties as the micelle sizes of fullerene polymer derivatives [22] and the degree of aggregation in aqueous solutions [23]. Thus, it was shown that the aggregation of fullerene derivatives affects their cytotoxicity [24,25], and the destabilization of lipid membranes [26]. Since these phenomena are also characteristic of fullerenols, it is necessary to investigate the relationship between their structure, their aggregation in aqueous solutions, and the influence of these parameters on their toxicity and biological activity. These studies are also of high importance because the problem of isolating pure regioisomeric fullerenols from a mixture of polyhydroxyfullerenes has not yet been solved. Fullerenols obtained by different methods, in addition to hydroxyl groups, can contain hemiacetal, carbonyl carbonyl, carboxyl, and keto-groups [27]. Therefore, fullerenols with the same number of hydroxyl groups obtained by different methods may exhibit different biological effects. 

The aim of the present work is to study the antiradical activity of fullerenol with an average molecular formula of C_60_(OH)_36_ in relation to the model stable free radical 2,2-diphenyl-1-picryl-hydrazyl (^•^DPPH) and ROS, such as superoxide O_2_^•−^, hydroxyl radicals ^•^OH, and singlet oxygen ^1^O_2_. Likewise, the work examines the fullerenol’s self-assembly in aqueous solutions and cell culture media, as well as its toxicity, protective effect on Madin–Darby canine kidney (MDCK) cell culture under UV irradiation, and antiviral effect against international reference strains of influenza subtypes A (H1N1)pdm09, A(H3N2), and B in vitro. Highly hydroxylated fullerenol C_60_(OH)_36_ in our investigation was used due to its high water solubility, which may have a positive effect on its biocompatibility.

## 2. Materials and Methods

### 2.1. Fullerenol Synthesis and Characterization 

Fullerene C_60_ with a purity of 99.9+ wt.% was synthesized at the Petersburg Nuclear Physics Institute named by B.P. Konstantinov of the National Research Center “Kurchatov Institute”. The data on the purity as well as chemical and crystal structures of fullerene C_60_ have been previously described in [28,29]. Fullerenol was synthesized by a two-step hydroxylation of fullerene C_60_ as described previously in [30] with some modifications. The presence of hydroxyl groups in fullerenol was confirmed using FTIR spectrometry in the 4000–400 cm^−1^ range on an IRTracer-100 spectrometer (resolution 4 cm^−1^, 32 scans) with an attenuated total reflectance (ATR) attachment (Shimadzu, Kyoto, Japan). The Raman spectroscopy was carried out on a Senterra II spectrometer (Bruker Optik GmbH, Ettlingen, Germany). The absorption spectra of fullerenol in water were obtained using UV-Vis spectrometry on an SF-2000 spectrophotometer (OKB Spectr LLC, St. Petersburg, Russia). Elemental analysis was performed on an SEM Tescan Vega 3 SBH (Tescan, Brno, Czech Republic) using the AdvancedAztecEnergy elemental composition determination system based on an X-act semiconductor energy-dispersive detector (Oxford Instruments, Abingdon, UK). The mass spectrum of fullerenol was obtained using the Ultraflextreme MALDI-TOF/TOF mass spectrometer (Bruker Daltonics, Bremen, Germany) in the reflector positive ion mode with a stainless-steel target. The α-cyano-4-hydroxycinnamic acid (CHCA, Bruker Daltonics, Bremen, Germany) was used as a matrix. The ^1^H NMR spectroscopy was carried out on a BrukerBioSpin AG Avance III HD 400 spectrometer (Bruker Corporation, Billerica, MA, USA). The solid-state ^1^H and ^13^C NMR spectra were obtained on a Bruker Avance III 400WB spectrometer (Bruker Corporation, Billerica, MA, USA). A two-channel sensor equipped with a magic angle sample rotation (MAS) system was used. The sample was rotated at a frequency of 12.5 kHz in a 4 mm zirconium oxide rotor. For excitation, a cross-polarization sequence (CP/MAS) was used with a 2 s relaxation delay time and 2 ms contact times; a sequence of direct excitation with decoupling from protons with a 30 s relaxation delay time and 3.2 μs of exciting pulse duration. Tetramethylsilane (TMS, Sigma Aldrich, St. Louis, MO, USA) was used as an external standard. The ESR spectrum was measured by a Bruker Elexsys E580 spectrometer (Bruker Corporation, Billerica, MA, USA) at room temperature. Thermal analysis (thermogravimetric analysis (TGA) and differential scanning calorimetry (DSC)) was carried out using a TGA/DSC 3+ apparatus (Mettler-Toledo AG, Greifensee, Switzerland) in a temperature range from room temperature to 1100 °C. The sample was heated at a rate of 10 °C/min in an argon atmosphere at a flow rate of 50 mL/min. 

### 2.2. Particle Size Distribution and ζ-Potential Measurements

Experiments were carried out to determine particle size distribution in fullerenol with concentrations of 100 and 200 μg/mL dissolved in deionized water, alpha modified Eagle’s minimum essential cell culture medium (α-MEM) with the addition of 10% fetal bovine serum (FBS, (Biolot, St. Petersburg, Russia)), and serum-free α-MEM cell culture medium (Biolot, St. Petersburg, Russia) after 24 and 72 h of incubation. The studies were performed using the dynamic light-scattering method on a Photocor Compact-Z analyzer (LLC Photocor, Moscow, Russia) with a laser wavelength of 654 nm at a scattering angle of 90° and a stabilized sample temperature of 25.0 °C. The size distributions of light-scattering particles based on their contribution to light scattering were obtained by analyzing the autocorrelation function of the intensity of light scattered by the samples using the regularization method in the DynaLS software (Vers. 2.9.1, Dr. Alexander Goldin, Alango Ltd., Tirat Carmel, Israel). The equipment and options of the Photocor Compact-Z analyzer allow measuring the ζ-potential of charged particles dissolved in the sample using laser Doppler anemometry. The analysis of the Doppler shift of the studied samples was carried out using the PALS (Phase Analysis Light Scattering) method, and the built-in software converted the data into the ζ-potential during measurement. The stability of the determined size distribution of light-scattering particles and the ζ-potential were determined after at least three measurements of each sample.

### 2.3. Radical Scavenging Activity of Fullerenol In Vitro

#### 2.3.1. DPPH Free Radical Scavenging Activity 

The antiradical activity of fullerenol against the stable free radical 2,2-diphenyl-1-picryl-hydrazyl (DPPH, extra pure, 95%, Sisco, Mumbai, India) was studied using UV-Vis spectrometry on an SF-2000 spectrophotometer (OKB Spectr LLC, St. Petersburg, Russia). A detailed description of the experiment was presented previously [29]. The antiradical activity of fullerenol ARA_DPPH·_ was also calculated at 5 and 30 min after the start of the ^•^DPPH reduction reaction using Equation (1):

(1) $${ARA}_{DPPH\cdot }=\frac{\left(A_{DPPH\cdot }-A_{sample}\right)}{A_{DPPH\cdot }}\cdot 100\%$$

where $A_{sample}$ and $A_{DPPH}$ are absorbance of 130 μM DPPH solutions in the presence and absence of 12.5–200 μg/mL fullerenol, respectively. All experiments were independently repeated six times.

#### 2.3.2. Hydroxyl Free Radical Scavenging Activity

The hydroxyl free radical scavenging activity of fullerenol was studied using spectrophotometric determination of 2,3- and 2,5-dihydroxybenzoic acids formed by the reaction of salicylic (2-hydroxybenzoic) acid with ^•^OH [31]. The hydroxyl radicals were generated during the Cr^6+^/Cr^3+^ reaction in the presence of H_2_O_2_, since fullerenol forms an insoluble complex with Fe^3+^ when the Fenton system is used to generate the hydroxyl radical [32]. A mixture of equal volumes of 5.0 mM solutions of salicylic acid (Sigma Aldrich, St. Louis, MO, USA) in ethanol (Vecton JSC, St. Petersburg, Russia), H_2_O_2_ (Vecton JSC, St. Petersburg, Russia), potassium dichromate (Vecton JSC, St. Petersburg, Russia) in deionized water, and an aqueous solution of fullerenol in concentrations of 12.5–200 μg/mL was incubated in a 37 °C water bath for 30 min. As a blank experiment, we replaced the potassium dichromate solution with water using a mixture similar to the working cuvette. The absorbance of samples of each concentration was measured at 312 and 330 nm wavelengths on an SF-2000 spectrophotometer (OKB Spectr LLC, St. Petersburg, Russia). The absorption of A_•OH_ was measured by replacing the fullerenol solution with an equal volume of deionized water. The experiments were repeated independently six times.

The scavenging activity (%) of the hydroxyl radical *ARA*_·OH_ was calculated using the following Equation (2):

(2) $${ARA}_{•OH}=\frac{\left(A_{•OH}-A_{sample}\right)}{A_{•OH}}\cdot 100\%$$

where $A_{•OH}$ and $A_{sample}$ are total absorption at 300 and 312 nm 30 min after the start of the reaction of the mixture of solutions of salicylic acid, H_2_O_2_, and potassium dichromate in the presence and absence of fullerenol, respectively.

#### 2.3.3. Superoxide Free Radicals Scavenging Activity 

The ability of fullerenols to scavenge superoxide radicals O_2_**^•−^** was determined by their degree of inhibition of the adrenaline autoxidation reaction. A detailed description of the experiment was presented previously [20]. It was found that fullerenol reacts not with the autoxidation products of adrenaline but with O_2_**^•−^**, which occurs during the intramolecular rearrangement of the adrenaline molecule in an alkaline environment in the presence of oxygen. The experiment was independently repeated six times.

The scavenging activity (%) of the superoxide radical anion ${ARA}_{O_{2˙}^{-}}$ was calculated using the following Equation (3):

(3) $${ARA}_{O_{2˙}^{-}=}\frac{\left(A_{O_{2˙}^{-}}-A_{sample}\right)}{A_{O_{2˙}^{-}}}\cdot 100\%$$

where $A_{O_{2˙}^{-}}$ and $A_{sample}$ are absorption 15 min after the start of the reaction of solutions in carbonate buffer (pH 10.7) of adrenaline hydrochloride (Moscow endocrine plant, Moscow, Russia) and a similar solution in the presence of fullerenol in concentrations of 12.5–200 μg/mL, respectively.

#### 2.3.4. Singlet Oxygen Scavenging Activity

The ability of fullerenols to scavenge singlet oxygen ^1^O_2_ was determined by the degree of its influence on the bleaching of 1,3-Diphenylisobenzofuran (DPBF, Leap Chem, Hong Kong, China), which is oxidized by ^1^O_2_. Singlet oxygen was generated by the Rose Bengal (RB, Sisco, Mumbai, India) photosensitization reaction. A detailed description of the experiment was presented previously [29]. The scavenging activity (%) of singlet oxygen was calculated using the Equation (4):

(4) ARAO21=Asample−AO21AO21·100%

where AO21 and $A_{sample}$ are absorption 8 min after the start of the oxidation reaction of DPBF and a similar solution in the presence of fullerenols at concentrations of 12.5–200 µg/mL, respectively. The experiments were independently repeated six times.

### 2.4. In Vitro Cytotoxicity Test and Protection of Cells Against UV Irradiation

We assessed the toxicity of fullerenol (incubated for 24 and 72 h) and its effect under UV irradiation against Madin–Darby canine kidney cells (MDCK) before studying their antiviral activity against the influenza A virus. The transplantable MDCK cell line was obtained from the collection of the Smorodintsev Research Institute of Influenza. The cells were cultured in α-MEM medium (Biolot, St. Petersburg, Russia) and supplemented with 10% FBS (Biolot, St. Petersburg, Russia) and antibiotics (penicillin and streptomycin (Biolot, St. Petersburg, Russia), and plated in 96-well plates (Thermo Scientific Nunc, Waltham, MA, USA) in an incubator in the presence of 5% CO_2_. Prior to adding fullerenol, the incubation medium was replaced with a serum-free α-MEM medium (Biolot, St. Petersburg, Russia). Serial dilutions of fullerenol (1.95–1000 μg/mL) were prepared in the serum-free α-MEM medium.

UV irradiation of the MDCK was carried out using a 400 W lamp (λ = 220–400 nm) (UFOB, Razryad JSK, Vladikavkaz, Russia) at a distance of 30 cm from the cell monolayer plate immersed in a cooling water bath. An external fan was also used for additional heat removal. Fullerenol at concentrations of 12.5–100 μg/mL was added for 1 h before UV irradiation. Then the cells were additionally incubated in an incubator in the presence of 5% CO_2_ for 18–20 h. To assess the effect of UV irradiation on the morphology of MDCK cells, micrographs of the cell monolayer were obtained using an inverted microscope, Leica DMI 1 (Leica Microsystems, Wetzlar, Germany) with a ×10/0.22 objective and an S40/0.45 condenser. In vitro cell viability was assessed using the standard MTT assay [33]. Absorbance was determined photometrically at 570 nm using a Varioscan plate analyzer (Thermo Scientific, Waltham, MA, USA). The data expressed as the dependence of absorbance at 570 nm on the fullerenol concentration decimal logarithm was used to calculate the 50% cytotoxic concentration (CC_50_) as well as the average effective concentration (EC_50_), which reduces cell viability by 50% UV-mediated cytotoxicity. To this end, dose–response curves were described using a 4-parameter model for a lognormal distribution (log(inhibitor) vs. Response Variable slope (four parameters)) in GraphPad Prism software (Vers. 8.0, GraphPad Software, San Diego, CA, USA).

### 2.5. In Vitro Anti-Influenza Activity of Fullerenol

The antiviral effect of fullerenol was determined against international reference strains of influenza virus subtypes circulating in the human population: A/California/07/2009(H1N1)pdm09, A/Cambodia/e08263/60/2020, and B/Austria/1359417/2021 (Victoria lineage). Before conducting the research, 1–2 passages of virus strains from the collection of the Smorodintsev Research Institute of Influenza were examined. Next, MDCK cells were infected with the virus diluted in increments of 10, with the maximum multiplicity of infection at 100TID_50_ (50% tissue infectious doses) per 100 μL. Fullerenol solutions in non-toxic concentrations (2.5–25 μg/mL) were added immediately before cell infection. Virus reproduction in the cell culture medium was measured by the microtechnique of hemagglutinating (HA-test) using a 0.75% chicken erythrocyte suspension according to the method recommended by the WHO [34]. The viral titer was expressed as lgTID_50_ per 100 μL. Antiviral activity was assessed by reducing the infectious viral titer (∆lg TID_50_) in the experimental well plates compared to the control (intact virus in 10-fold dilutions). The boundary criterion for assessing antiviral activity was a decrease in the infectious viral titer by at least 2lgTID_50_ (∆lg TID_50_ ≥ 2). Since the degree of cell viability inhibition in cell culture correlates with the development of viral infection in vitro, the degree of the cytopathogenic effect (CPE) of the virus on cells was also assessed using the standard MTT assay, as described above. The average virus inhibitory concentration of fullerenol (IC_50_), causing the inhibition of 50% of viral activity, was calculated using a 4-parameter model for a lognormal distribution (log(inhibitor) vs. Response Variable slope (four parameters) in GraphPad Prism software (Vers. 8.0, GraphPad Software, San Diego, CA, USA). Also, as a criterion of antiviral effectiveness, the selectivity index (SI) was calculated. The SI is the ratio of the average cytotoxic concentration CC_50_ to the average inhibitory antiviral concentration (IC_50_).

### 2.6. Statistical Analysis

All biological experiments were repeated at least six times. Statistical analysis was performed using GraphPad Prism software (Vers. 8.0, GraphPad Software, San Diego, CA, USA). The data were analyzed using an unpaired Student’s *t*-test to identify significant differences between the fullerenol-treated cells and the intact control cells. The differences from the control were considered significant at a significance level of less than 0.05. Calculations of the mean and SD of antiradical activity were performed using Origin software (Vers. 9.2, Origin Lab Corporation, Northampton, MA, USA). The data were presented as the mean (six independent experiments) ± S.D.

## 3. Results

### 3.1. Characterization of Fullerenol

**Figure 1:**
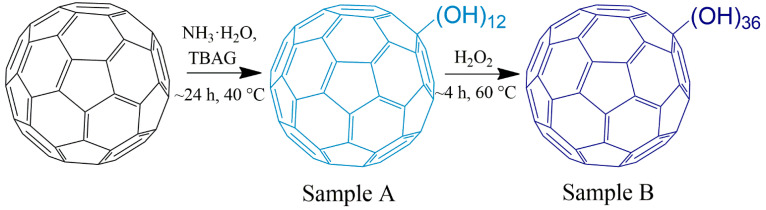
A schematic representation of fullerenol synthesis.

Figure 1 shows the process of two-stage hydroxylation of fullerene C_60_. This procedure was described earlier [30] and is the formation of low-hydroxylated fullerenol in the first stage of the process and its subsequent hydroxylation by treatment with hydrogen peroxide in the second stage. We have experimentally established that by varying the concentration of peroxide, it is possible to obtain fullerenols with different degrees of hydroxylation. In this investigation, a solution of 20 wt.% of hydrogen peroxide was used to obtain fullerenol with 36 hydroxyl groups. Sample A was found to be insoluble in water, while Sample B had a solubility of ~60 mg/mL.

**Figure 2:**
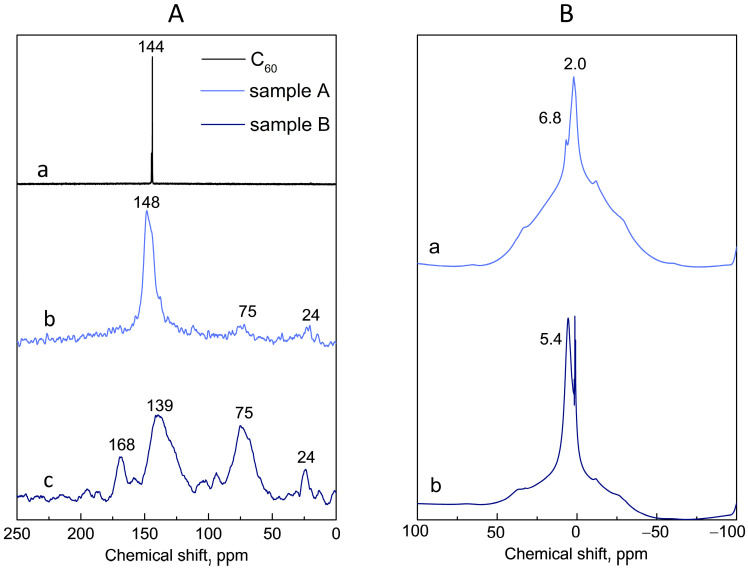
(**A**) Solid-state ^13^C-NMR spectra of fullerene C_60_ (a), Sample A (b), and Sample B (c). (**B**) ^1^H Solid-state ^1^H-NMR spectra of Sample A (a) and Sample B (b).

The chemical shift of unmodified sp^2^-hybridized carbon atoms of fullerene C_60_ corresponds to a value at 144 ppm [27], as can be seen in Figure 2A, curve (a). The narrow well-resolved signal in the ^13^C solid-state NMR spectrum of fullerene C_60_ appears due to the rapid rotation of the fullerene molecules and the effective averaging of the dipole–dipole interactions of the magnetic moments of the carbon atoms, as in the case of liquid-state NMR. As shown in Figure 2A (curves b and c), several signals are observed for Samples A and B. The fullerene modification with hydroxyl groups leads to a substantial broadening of the signal at 144 ppm due to the significant inhomogeneity of the electronic structure of fullerenol associated with the redistribution of sp^2^- and sp^3^-hybridized carbons [35,36]. The ^13^C CP MAS NMR spectra of Samples A and B contain signals at 75 and 25 ppm, which correspond to sp^3^ carbons with singly attached OH groups [27,37,38,39] and carbon (C–H) adjacent to the hydroxyl group [40], respectively. Sample B may also contain a small amount of pair-grafted hydroxyls, the signal of which at about 80 ppm [35] overlaps with a broad maximum at 75 ppm. The appearance of a signal in the spectrum of Sample B at 168 ppm may indicate the presence of a small number of carboxyl groups [39,41], which can be pH-dependently reversibly formed in fullerenols [27].

The maximum of about 2.0 ppm in the ^1^H solid-state NMR spectrum of Sample A (Figure 2B, curve a) can be attributed to the signal of the proton of single attached OH groups, about 7 ppm to the proton attached to the carbon adjacent to the hydroxyl group. The ^1^H liquid-state NMR spectra (Figure S1) of Sample A contain signals corresponding to both the protons of pair-bonded hydroxyls (signals around 1.2 ppm) and signals corresponding to the case when the hydroxyl group shares a bond with a proton (OH group protons in the range from 1.8 to 2.5 ppm, the neighboring proton in the range from 4 to 7 ppm) [35]. At the same time, in the ^1^H solid-state NMR spectrum of Sample B (curve b in Figure 2B), the narrow lines at 0.93 ppm and 1.3 ppm can also be either a useful signal or artifact lines from the water adsorbed on the rotor surface.

**Figure 3:**
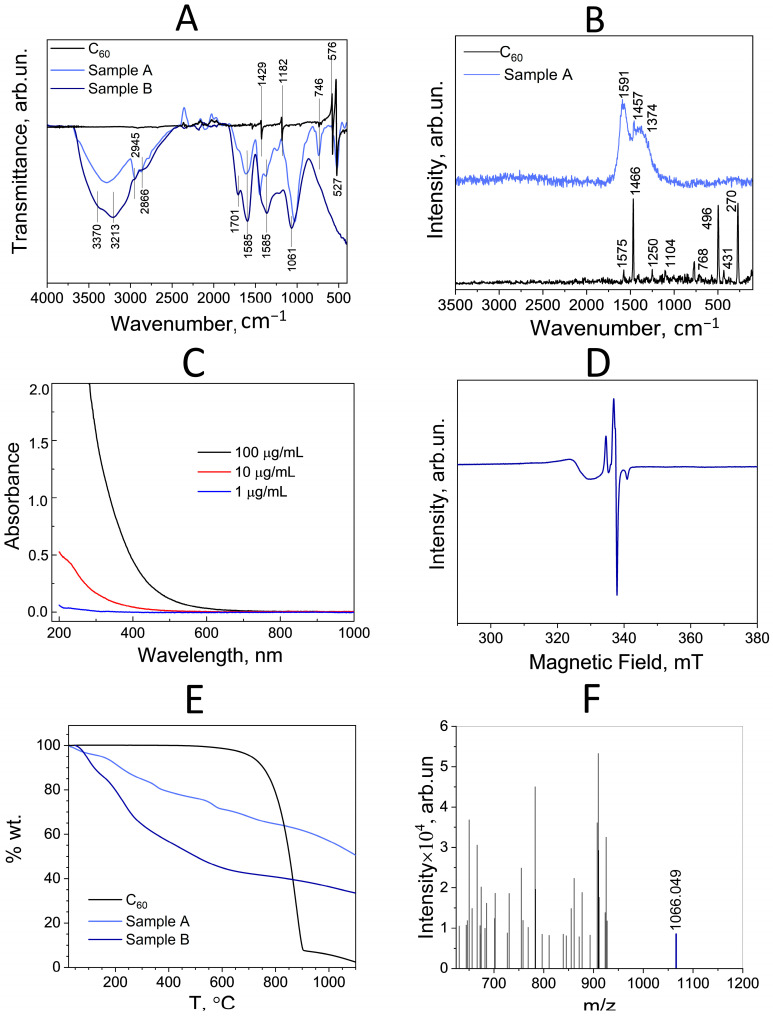
(**A**) FTIR spectra of fullerene C_60_ and Samples A and B. (**B**) Raman spectra of pristine C_60_ and Sample A. (**C**) Absorbance spectra of Sample B in aqueous solutions. (**D**) ESR spectrum for solid-state Sample B at 298 K. (**E**) TGA curves of fullerene C_60_ and Sample A and B obtained at 10 °C/min heating rate under Ar atm. (**F**) MALDI-MS profile of Sample B and matrix effect of the CHCA.

The FTIR spectrum of Sample B (Figure 3A) did not contain four intramolecular characteristic vibration modes of fullerene C_60_ at 527 cm^−1^, 576 cm^−1^, 1182 cm^−1^, and 1429 cm^−1^ [42] bands of the characteristic. The presence of hydroxyl groups attached to the fullerene molecule was confirmed by the appearance of strong bands at 1062 cm^−1^, 1361 cm^−1^, 1720 cm^−1^, and 2927 cm^−1^, which correspond to the υ C–O, δ_s_ C–O–H, υ C = C, and C-H stretching modes [43,44,45,46]. A small peak at ~1700 cm^−1^ corresponds to a carboxyl group, which can be formed by oxidation of the hydroxyl group [27], which agrees with ^13^C solid-state NMR data. A broad, intense band in the region of 3300 cm^−1^ corresponds to the vibrations of the –O–H bonds of hydroxyl groups that are predominantly linked by hydrogen bonds [47] and water from the hydration shell. 

The Raman spectrum of pristine C_60_ (Figure 3B) contains the characteristic Raman modes for fullerene at room temperature [48]. On the Raman spectrum of Sample A, almost all bands characteristic of pristine C_60_ disappear due to the insertion of functional groups onto the C_60_ surface, which reduces the symmetry of the molecule. The Raman spectrum of Sample A displayed two broad peaks at 1374 cm^−1^ and 1591 cm^−1^, consistent with the D- and G-bands of C–C on the carbon cage of fullerenol [49,50]. It was not possible to obtain a satisfactory Raman spectrum of Sample B, since it luminesced when excited by all available laser wavelengths (532 and 785 nm).

The absorption spectra of fullerenol in deionized water in the UV-Visible region (Figure 3C) did not contain characteristic maxima due to the significantly reduced π-conjugation compared to fullerene C_60_ [43].

The fact that the structure of fullerenol is not limited to the presence of hydroxyl groups is also evidenced by the ESR spectrometry data. The ESR spectrum of Sample B (Figure 3D) shows a clear signal (g = 2.00307) when measured at room temperature in the solid state, which is consistent with the results obtained earlier [43,51]. The paramagnetic behavior of fullerenol may be due to the presence of a stable cyclopentadienyl radical in the molecule. At the same time, Sample A did not exhibit paramagnetic behavior.

The stepwise process of dehydration and subsequent dehydroxylation of fullerenol is clearly visible in the curves corresponding to TGA (Figure 3E). In the temperature range from room temperature to 130 °C, the mass loss of fullerenol is associated with the loss of secondary water, which is associated with hydroxyl groups through hydrogen bonds [43]. In the temperature range from 130 °C to 570 °C, the loss of mass is associated with the dehydroxylation process, the formation of hemiketals, and the rearrangement of hydroxyl groups according to the pinacol type and their degradation. It was also supported by the broad endotherm peak at 300 °C on DSC curves of Sample A and B (Figure S2). The mass loss at temperatures above 570 °C is associated with fullerene cage decomposition. The number of fullerenol hydroxyl groups (*n*) of Sample A and Sample B was estimated according to Equation (5) [52]:

(5) $$n\approx \frac{M\left(C_{60}\right)}{y}\times \frac{x}{M\left(OH\right)}$$

where *x* is the mass loss (in wt%) in the 130–570 °C temperature range, corresponding to the removal of hydroxyl groups; *y* is the remaining mass (in wt%) at 570 °C, corresponding to dehydroxylated fullerene; and M(C_60_) and M(OH) are the molecular weight of fullerene and hydroxyl groups, respectively.

According to Equation (5), using the mass loss in the temperature range from room to 130 °C and the molecular weight equal to eighteen as *x*, the number of water molecules in the hydration shell of Sample A and Sample B was also determined. As shown in Table 1, thermal analysis data are consistent with the results of elemental analysis, considering that under the vacuum created during elemental analysis, water is removed from the hydration shell of fullerenols at room temperature. At the same time, we did not detect a peak corresponding to the molecular ion of fullerenol in the MALDI-mass spectrum of Sample A (Figure 3F). Probably, dehydroxylation of fullerenol occurred during laser ionization of the sample. 

Thus, the intermediate product of the synthesis, Sample A, was low-hydroxylated fullerenol C_60_(OH)_12_, and the final product, Sample B, was highly hydroxylated fullerenol with an average molecular formula of C_60_(OH)_36_.

Further, in the experiments on studying self-assembly in solutions, antioxidant activity, and all biological experiments, the highly hydroxylated fullerenol C_60_(OH)_36_ sample was used.

### 3.2. Self-Assembly of Fullerenols in Aqueous Solutions and Cell Cultural Media 

It is well known that water-soluble fullerene derivatives easily aggregate and form clusters in aqueous solutions [45,46]. The self-assembly of fullerenols is the result of the simultaneous occurrence of two processes in aqueous solutions: hydrophilic interactions due to hydrogen bonds arising between the fullerenol hydroxyl groups and water molecules, as well as intermolecular hydrophobic interactions arising due to the uneven distribution of hydroxyl groups on the fullerene surface. The process of the self-assembly of fullerenols may depend on the differences in electron affinity caused by the peculiarities of the arrangement of hydroxyl groups on the fullerene surface [48], on the properties of the solvent, such as pH [53,54] and temperature [54], and on the type of cell culture medium [55]. In the present work, we studied the self-assembly of fullerenols at concentrations of 100 and 200 μg/mL. Fullerenols were incubated for 24 and 72 h in deionized water, as well as in α-MEM cell culture medium with the addition of 10% FBS and a serum-free α-MEM medium. The hydrodynamic radii of fullerenol particles are presented in the mass distribution (Table 2) and in the distribution by contribution to light scattering (Figure S2).

Aqueous solutions of fullerenol C_60_(OH)_36_·10H_2_O (Figure S3A,B) can be considered as polydisperse suspensions containing two types of particles. Such a bimodal distribution was previously observed for fullerenol dispersions obtained by different methods [46,48]. With longer incubation, a shift in distribution of both fractions toward larger formations was observed (Figure S3), except for the smallest aggregates in water at the minimum studied concentration of fullerenol (Figure S3). The relative stability of aqueous solutions of fullerenols is evidenced by the fact that the absolute value of the ζ-potential with increasing incubation time (insets in Figure S3A,B) exceeded the value of ~30 mV, which is considered the threshold of stability for colloidal systems. The shift of both fractions in all distributions toward larger sizes is clearly greater in more concentrated samples. This may be because fullerenol clusters in less concentrated aqueous solutions are surrounded by a well-organized layer of water molecules connected by hydrogen bonds (a stable hydrophilic shell), which prevents interaction with neighboring molecules [56].

However, it is equally important to evaluate the behavior of fullerenols in cell culture media in which in vitro biological experiments are carried out. The size of the particles can influence their cytotoxicity and plays an important role in their interaction with biological objects and their ability to penetrate the cell membrane [24]. In addition, the protein present in the cellular environment can interact with fullerenols, affecting their hydrophobicity, reactivity, and ζ-potential [55]. As can be seen from the size distributions of fullerenol particles, the shifts in fractions are minimal in the culture medium depending on the incubation time and are practically independent of the fullerenol concentration for samples with the addition of serum (Figure S2C,D). However, in solutions of fullerenols in a serum-free medium (Figure S2E,F), all distributions are significantly shifted toward larger sizes compared to serum-containing solutions. The authors also noted the precipitation of fullerenol in serum-free α-MEM medium after 72 h of incubation. This result is consistent with the data obtained in [24], where it was shown that fullerenol C_60_(OH)_22–24_ after 12 h of incubation was much more stable in a cell culture medium with 10% FBS, whereas in serum-free medium, it aggregated within a few hours, as confirmed by DLS and ζ-potential measurements. Srdjenovic et al. [55] measured the particle size distribution and ζ-potential of fullerenol C_60_(OH)_24_ dissolved in deionized water (pH 6) and cell culture medium with 10% FBS (24 h incubation at 37 °C in the dark). Fullerenol C_60_(OH)_24_ nanoparticles in an aqueous solution generally had sizes in the range of 2–8 nm, while in a medium containing FBS, fullerenol formed polydisperse aggregates with sizes of 2–30 nm. The ζ-potential values of C_60_(OH)_24_ in an aqueous solution were significantly lower (–58 mV) compared to the ζ-potential value of fullerenol in RPMI + 10% FBS (–7.9 mV). 

It should be noted that the average hydrodynamic radii of fullerenol C_60_(OH)_36_·10H_2_O particles that form the basis of these solutions (>80–99% in distribution by mass) during 24 h incubation does not exceed 100 nm in aqueous solutions and cell culture media. This size of nanoparticles is favorable for in vivo application [57].

### 3.3. Fullerenol Radical Scavenging Activity In Vitro

**Figure 4:**
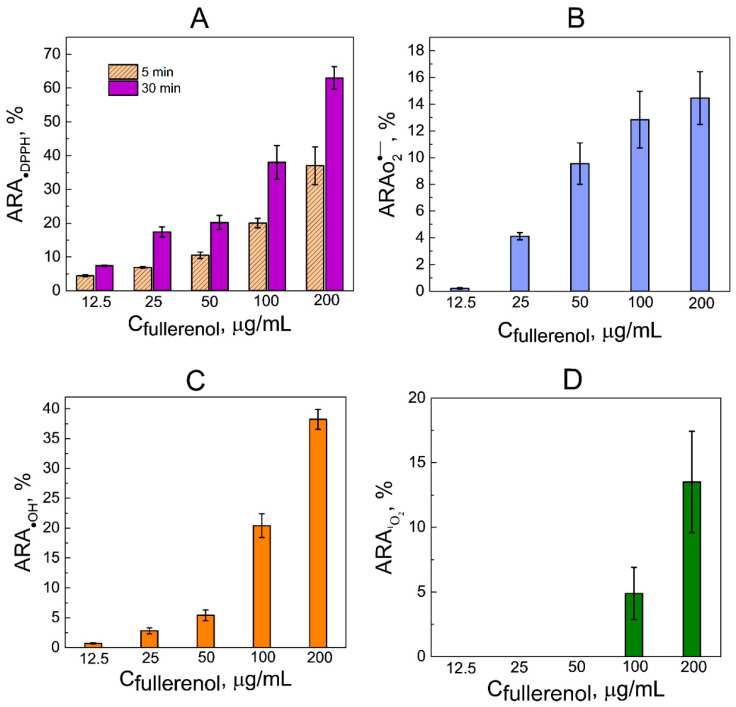
Radical scavenging activity of fullerenol. (**A**) DPPH radicals scavenging activity. (**B**) Superoxide radicals scavenging activity. (**C**) Hydroxyl radical scavenging activity. (**D**) Singlet oxygen scavenging activity. The data are presented as mean values ± SD (*n* = 6 independent experiments). In (**A**–**D**), the difference between the control and the experiments is reliable (*p* < 0.05).

As can be seen in Figure 4, the activity of fullerenol C_60_(OH)_36_·10H_2_O against DPPH, superoxide, hydroxyl radicals, and singlet oxygen has a concentration dependence, which is consistent with the data known for fullerenols with varying degrees of hydroxylation and fullerenol nanocomposites [15,17,58,59,60,61,62]. At the same time, fullerenols at concentrations below 100 μg/mL did not exhibit antioxidant activity against singlet oxygen (Figure 4D).

### 3.4. Fullerenol Toxicity In Vitro

**Figure 5:**
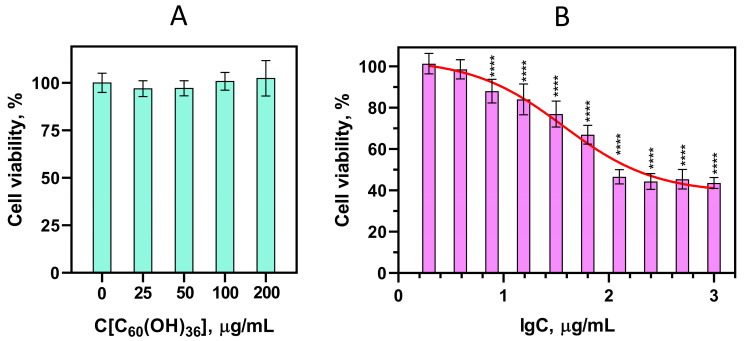
The viability of MDCK cells incubated with C_60_(OH)_36_ for 24 h (**A**) and 72 h (**B**) obtained by MTT assay. The results are mean values ± SD (*n* ≥ 6 independent experiments). In (**A**), the difference between the control and the experiments is unreliable (*p* ≥ 0.05). In (**B**) nonlinear approximation, 4-parameter model (GraphPad Prism Software Vers. 8.0). **** *p* < 0.0001.

Fullerenol C_60_(OH)_36_ at concentrations up to 200 μg/mL had no cytotoxic effect on MDCK when cells were incubated in the presence of fullerenols for 24 h in a serum-free α-MEM (Figure 5A). These data are consistent with the results we obtained earlier while incubating fullerenols with a lower degree of hydroxylation C_60_(OH)_30_ that were obtained by the same method [30]. However, when MDCK was incubated for 72 h in the presence of fullerenol C_60_(OH)_36_, its cytotoxic effect was detected (Figure 5B). The average cytotoxic concentration reducing cell viability by 50% (CC_50_) was 40.00 ± 1.13 μg/mL (30.0 ± 1.0 μM). In addition, when cells were incubated in the presence of fullerenol at concentrations of 50–200 μg/mL in a serum-free α-MEM for 72 h, a partial precipitation of the compound in the cell culture medium was noted, while no such effect was noted in deionized water. This result is consistent with the DLS data obtained in the present work, as well as with the results obtained in [24,55] where it was shown that fullerenol C_60_(OH)_x_ was much more stable in the cell culture medium with serum and formed clusters of smaller sizes compared to the serum-free cell culture medium in which it aggregated after just a few hours.

It should be noted that the cytotoxic effect of fullerenol obtained during 72 h incubation may be useful in anticancer drug applications. Thus, Guo et al. showed that fullerenol C_60_(OH)_20±2_ at a concentration above 160 μM induced apoptosis in K562 chronic myeloid leukemia cells [63].

### 3.5. Cytoprotective Effect of Fullerenol Against UV Irradiation

**Figure 6:**
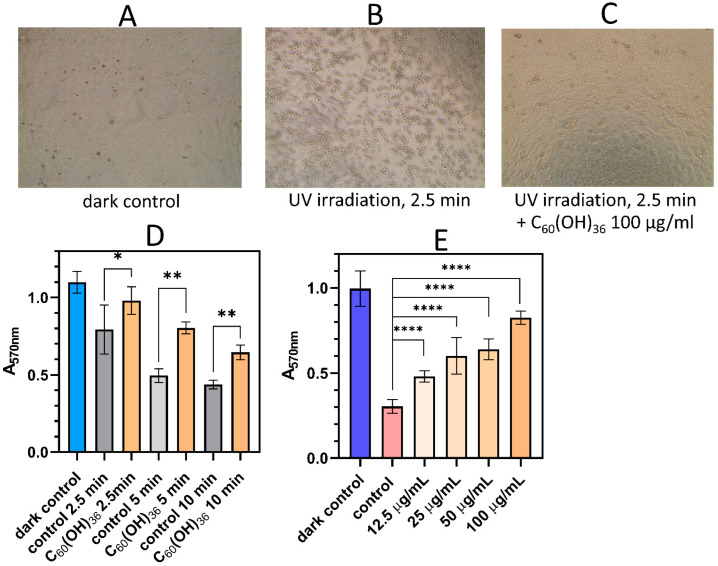
Protective effects of fullerenol against UV irradiation. (**A**) Morphology of MDCK cells without fullerenol in dark control. (**B**) Morphology of MDCK cells after UV irradiation for 2.5 min. (**C**) Morphology of MDCK cells under UV irradiation for 2.5 min in the presence of 100 μg/mL fullerenol. (**D**) Protective effect of fullerenol on cells in culture against UV phototoxicity. Fullerenol concentration is 50 µg/mL, and irradiation time is 2.5 ÷ 10 min. After irradiation, the cells were incubated for 18–20 h in the dark. * *p* < 0.05; ** *p* < 0.001 (**E**) Effect of fullerenol concentration on UV-mediated toxicity in vitro. Irradiation time: 7 min. **** *p* < 0.0001. Values are expressed as the mean ± S.D. (*n* ≥ 6).

Upon UV irradiation of intact MDCK cells in the culture medium, a linear increase in phototoxicity was observed with increasing exposure time (Figure 6D, control). Fullerenol showed a cytoprotective effect at all the studied irradiation times (Figure 6D). The morphology of MDCK cells exposed to UV irradiation for 2.5 min in the presence of fullerenol at a concentration of 100 μg/mL (Figure 6C) was practically no different from the dark control (Figure 6A), while after UV irradiation of MDCK untreated with fullerenol, an almost complete destruction of the cell monolayer occurred, and the cells rounded and detached from the substrate (Figure 6B). The cytoprotective effect of fullerenol C_60_(OH)_36_·10H_2_O upon UV irradiation had a pronounced dose-dependent nature, as shown in Figure 6E. The average effective concentration (EC_50_) of fullerenol, which reduces UV-mediated toxicity by 50% when irradiated for 7 min (the irradiation time during which the damaging effect of UV light is already obvious but is not sufficient to destroy cells completely), was 39.6 ± 1.1 μg/mL (~29.7 ± 1.0 μM). It should be emphasized that during the 24 h incubation period, the viability of MDCK treated with fullerenol C_60_(OH)_36_ at a concentration corresponding to EC_50_ did not differ significantly from the control (Figure 6A).

### 3.6. Fullerenol Anti-Influenza Activity In Vitro

**Figure 7:**
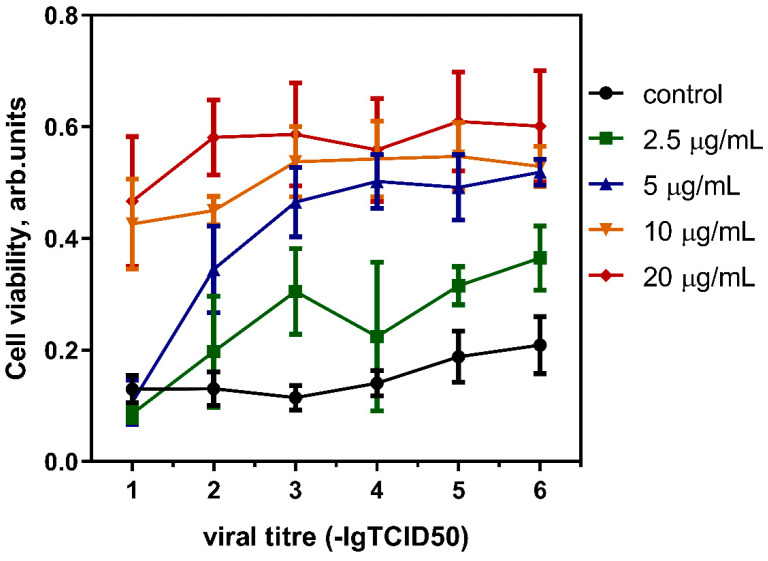
Activity of C_60_(OH)_36_·10H_2_O against the A/California/07/2009 (H1N1)pdm09 reference strain, measured by the MTT test. The results are mean values ± SD (*n* ≥ 6 independent experiments).

The results of the study of the antiviral activity of fullerenol against the A/California/07/09 (H1N1)pdm09 strain by the MTT assay are presented in Figure 7. The antiviral effect was expressed in a decrease in the cytopathogenic effect of the virus and an increase in cell viability (integral activity of respiratory enzymes) in the presence of the virus in 10-point dilutions from 1 to 6. In accordance with the obtained SI values, it turned out that fullerenol C_60_(OH)_36_·10H_2_O exhibited moderate antiviral activity against influenza A(H1N1) and A(H3N2) viruses. If the IC_50_ is lower than the CC_50_, the virus will be neutralized before causing damage to host cells, and they will not experience any side effects when treated with fullerenol.

Data assessing the antiviral effect of fullerenol against three influenza subtypes circulating in the human population are presented in Table 3.

Fullerenol was effective against both subtypes of influenza virus A with a satisfactory SI (perspective preparations are assumed to have an SI ~10 in vitro [64]. At the same time, influenza virus B of the Victoria lineage (now circulating in the human population) did not reach the generally accepted minimum criterion of antiviral activity (ΔlgTID_50_ ≥ 2.0) even at the highest non-toxic concentration (25 μg/mL).

## 4. Discussion

The currently proposed mechanisms of the antiradical activity of fullerenols [65] have not been reliably confirmed experimentally, and there is no consensus on which of the reaction centers—hydroxyl groups or double bonds—determine the antiradical activity of fullerene derivatives to a greater extent. For example, it is assumed that the mechanism of removal of hydroxyl radicals in the presence of fullerenols depends on the degree of their hydroxylation. In solutions of fullerenols with a low degree of hydroxylation, the preferred mechanism is the addition of ^•^OH to the sp^2^-carbon on the fullerene cage, whereas in the presence of highly hydroxylated fullerenols, the most likely mechanism is the capture of hydroxyl radicals by the proton of the hydroxyl group [64]. In all likelihood, the removal of free radicals in the presence of fullerenols occurs in a more complex way because of their interaction with reaction centers, whose availability depends on the nature of the self-organization of fullerenols in solutions. Volkov et al. [66] showed that the structure of fullerene C_60_ amino acid derivatives does not affect their antiradical properties. According to the authors, these properties are only determined by the effective total surface area of nanoparticles of fullerene derivatives and increase with decreasing their size. This surface can be characterized as a nanowall on which radical death occurs. This assumption can also be applied to fullerenol: due to hydrogen bonds, the polyhydroxylation of the fullerene framework can lead to the formation of larger aggregates surrounded by a hydration shell, which forms a monolayer [67], impeding the diffusion of radicals to reaction centers. Consequently, fullerenols with low and medium degrees of hydroxylation would have greater antiradical activity in this case. At the same time, fullerene derivatives with a greater number of hydrogen bonds between functional groups had higher activity against O_2_^•−^ [68]. Taking into account the data obtained in our study on the dependence of fullerenol aggregation on the type of solution, further study of the effect of fullerenol aggregation on its antioxidant and antiviral activity is necessary. In addition, the study conducted in this paper is limited to assessing only short-term stability of fullerenols in aqueous solutions and biological media.

Even if the mechanisms of antiradical activity are not yet entirely understood, the cytoprotective effect of fullerenol C_60_(OH)_36_ in vitro that has been established in this work can be attributed to its ability to react with ROS generated in the culture medium under UV irradiation. Thus, the cytoprotective effect of fullerenols with varying degrees of hydroxylation on human skin, the keratinocyte cells (HaCaT) of the human skin under UV irradiation, was previously demonstrated [14]. The damage caused by UV irradiation to HaCaT was significantly suppressed in the presence of fullerenols C_60_(OH)_32–34_·7H_2_O and C_60_(OH)_44_·8H_2_O. The cytoprotective effect of C_60_(OH)_44_·8H_2_O was superior to that of C_60_(OH)_32–34_·7H_2_O and was more significant when irradiated with UVB light compared to UVA irradiation. The authors showed that the effects mediated by the UVB irradiation of HaCaT cells, such as an increase in the level of intracellular oxidative stress, the formation of cyclobutene-pyrimidine dimers, and chromatin condensation, were suppressed by C_60_(OH)_44_·8H_2_O due to its absorption of ROS. A similar result was presented in [15], where fullerenol was shown to restore damage to UVB-irradiated human corneal epithelial cells (hCECs) by reducing the level of cellular oxidative stress and increasing cell proliferation, thus realizing a decrease in apoptosis. The protective effect of fullerenol on cells exposed to X-ray irradiation and the reduction in the manifestations of radiation dermatitis are also associated with their activity against ROS [69]. However, there are also some contradictions: In [70], it was established that during UVA irradiation of fullerenol C_60_(OH)_24_ dissolved in D_2_O, singlet oxygen and superoxide are generated. In this case, phototoxicity upon irradiation of HaCaT cells in the presence of fullerenol was mainly due to the superoxide anion radical.

The antiviral effect of fullerenols may also be associated with their antioxidant activity. Thus, it was previously shown that the antiviral activity of fullerenols C_60_(OH)_12–14_, C_60_(OH)_18–24_, and C_60_(OH)_30–38_ depends on the number of hydroxyl groups, with the maximum activity found in fullerenol with an average degree of hydroxylation C_60_(OH)_18–24_ [71]. However, in all likelihood, the antiviral effect of fullerenols is not limited to their antioxidant effect. Zaremba et al. [72] studied the activity of polyhydrate fullerenes with a mass ratio of 78.1% C_60_/C_70_ and 21.9% C_76_/C_78_/C_84_ on the influenza A virus (H1N1). It has been shown that the mixture of fullerenols, along with their low toxicity, exhibits high antiviral activity with a reduction in the infectious titer of the virus by up to four orders of magnitude. In addition, the studied fullerenols did not affect the hemagglutination process and did not exhibit significant preventive activity. The authors suggested that the high antiviral effect of polyhydrate fullerenes on the influenza A virus is associated with their interaction with the viral RNA polymerase. Additionally, for thiosialoside-functionalized fullerenes [73], it was shown that they do not inhibit the hemagglutination of erythrocytes at the maximum tested concentration of 50 μM, but at the same time they moderately inhibit the neuraminidase of influenza virus strains A(H1N1) (A/PR/8/34 A/Virginia/ATCC3/2009), H3N2 (A/HK/7/87), and influenza type B (B/HK/5/72). Shoji et al. [74] identified fullerene derivatives that inhibited the endonuclease activity of the N-terminal PA domain or the full-length PA protein of influenza A virus RNA polymerase in vitro. An in silico docking simulation analysis showed that fullerenes can bind to the active pocket of the PA endonuclease. The absence of the antiviral effect of fullerenol C_60_(OH)_36_ on the influenza B virus established in this study can be explained by a significant difference in the structure of the matrix proteins that form ion channels in influenza A and B viruses [75].

## 5. Conclusions

The results of the presented study are important for understanding the biological activity of fullerene derivatives. Even though the mechanisms of the antioxidant, cytoprotective, and antiviral effects of fullerenols have not yet been clearly established, the fact that they quite effectively absorb free radicals, including those mediated by UV irradiation, indicates that fullerenols have great potential as a broad-spectrum base drug for use in diseases accompanied by an undesirable increase in the amount of ROS. This effect, combined with the fact that fullerenol showed moderate activity against influenza A viruses, indicates that fullerenols may be promising as antiviral drugs with nonspecific effects that prevent the development of resistance.

## Figures and Tables

**Table 1:** Hydroxyl group determination of Samples A and B.

Sample	Weight Loss, wt.%		Elemental Analysis Data, C:O, wt.%	Average Formula
	rt-130 °C	130–570 °C		
**A**	4.4	21.7	81.6 ± 1.5:18.4 ± 1.3 (78.9:21.1) *	C_60_(OH)_12_·2H_2_O
**B**	11.27	41.6	56.4 ± 1.3:43.6 ± 2.0 (54.1:45.9) *	C_60_(OH)_36_·10H_2_O

* Values in brackets are calculated data.

**Table 2:** Distribution by mass of hydrodynamic radii* of a fullerenol particle.

C_Fullerenol_, μg/mL	Incubation Time, h	H_2_O	Cell Culture Medium	
			α-MEM + 10% FBS	α-MEM
100	24	44 ± 7 (>99%) 204 ± 40 (<1%)	17 ± 3 (~99%) 67 ± 8 (~1%)	68 ± 4 (>99%)
	72	45 ± 6 (>99%) 255 ± 40 (<1%)	25 ± 8 (~99%) 110 ±25 (~1%)	108 ± 11 (~98%) 490 ± 50 (~2%)
200	24	31 ± 4 (~80%) 135 ± 21 (~20%)	19 ± 4 (~98%) 88 ± 15 (~2%)	60 ± 12 (~97%) 416 ± 70 (~3%)
	72	64 ± 20 (~99%) 400 ± 60 (<1%)	36 ± 6 (>99%) 110 ±20 (<1%)	136 ± 20 (>99%)

**Table 3:** Antiviral effect in vitro of fullerenol C_60_(OH)_36_·10H_2_O on the human influenza viruses.

Human Influenza Subtype	IC_50_, μg/mL		Medium SI
	Hemagglutination Assay	MTT Assay	
A(H1N1)pdm09	4.0 ± 1.9	3.9 ± 1.8	9.9 ± 4.6
A(H3N2)	3.6 ± 0.4	2.6 ± 0.2	12.5 ± 1.3
B	Minimum criterion of antiviral effect not achieved		

## Data Availability

Data are contained within the article or Supplementary Materials.

## Supplementary Materials

The following supporting information can be downloaded at: https://www.mdpi.com/article/10.3390/antiox13121525/s1, Figure S1. ^1^H-NMR spectra of Sample B in D_2_O; Figure S2. Differential scanning calorimetry (DSC) curves of fullerene C_60_ (a) and Sample A (b) and Sample B (c) obtained at 10 °C/min heating rate under Ar atm. Figure S3. Fullerenol size distributions by intensity (200 and 100 µg/mL) after 24 and 72 h from the fullerenol incubation time in water (A,B), α-MEM with 10% FBS (C,D), and a α-MEM serum-free (E,F) media. The inserts (A,B) show the ζ-potential ± SD of fullerenol in an aqueous solution. The measurements were carried out at 25 °C. The mean hydrodynamic radii and SD values of the analyzed particles were obtained by the DynaLS software (Vers. 2.9.1, Dr. Alexander Goldin, Alango Ltd., Tirat Carmel, Israel).
