# Supplementary material for: Fullerenol C60(OH)36: Antioxidant, Cytoprotective, Anti-Influenza Virus Activity, and Self-Assembly in Aqueous Solutions and Cell Culture Media
Source: Antioxidants (Basel). 2024 Dec 13;13(12):1525. doi: 10.3390/antiox13121525 (PMC11727559; doi:10.3390/antiox13121525)
Supplement: Supplementary file 1 [file antioxidants-13-01525-s001.zip › antioxidants-3363870-supplementary.pdf]

Supplementary Materials

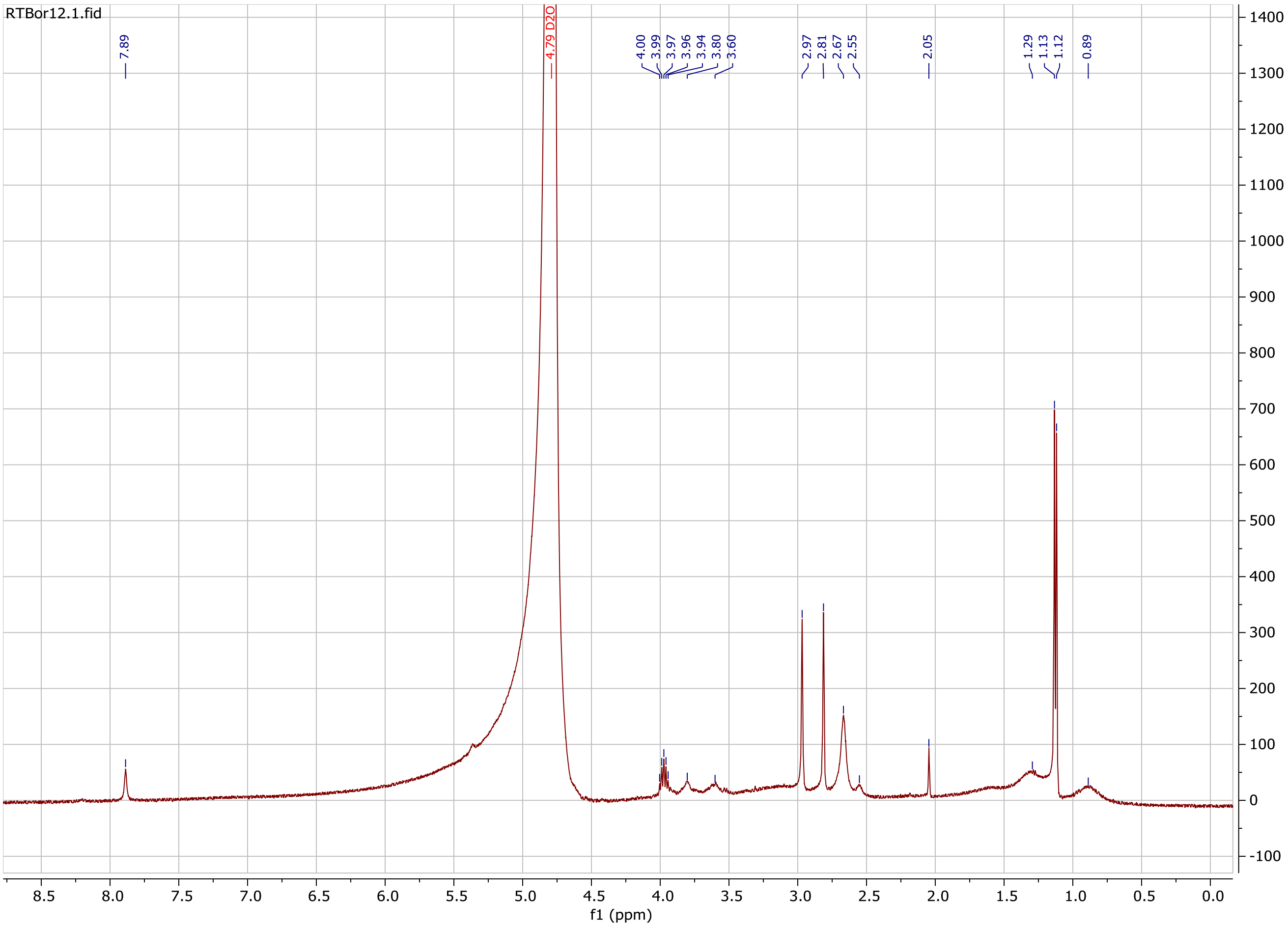

Figure S1. ^{1}H-NMR spectra of Sample B in D_2O

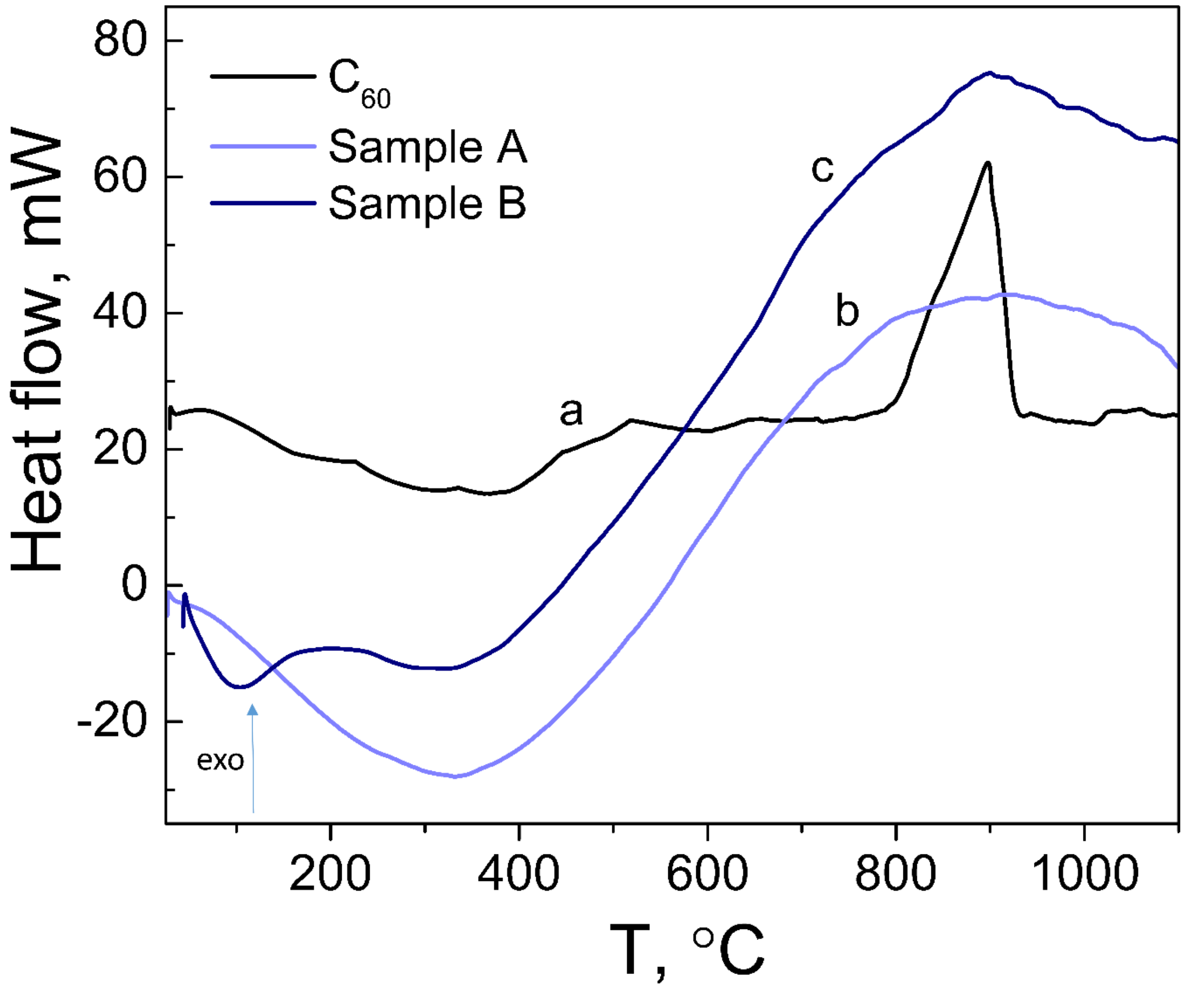

Figure S2. Differential scanning calorimetry (DSC) curves of fullerene C_{60} (a) and Sample A (b) and Sample B (c) obtained at 10 °C/min heating rate under Ar atm.

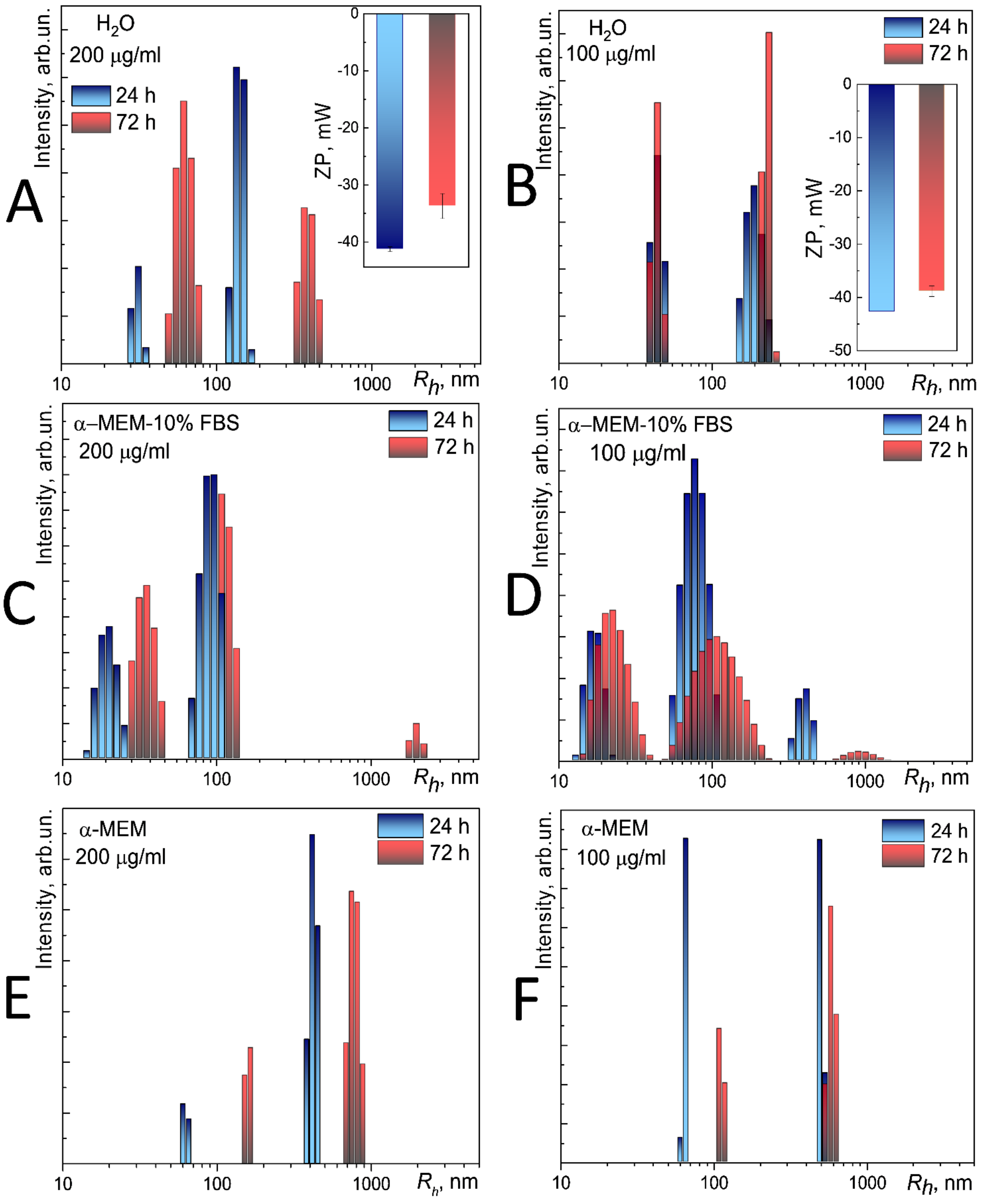

Figure S3. Fullerenol size distributions by intensity (200 and 100 μg/mL) after 24 and 72 h from the fullerenol incubation time in water (A,B), α-MEM with 10% FBS (C,D), and a α-MEM serum-free (E,F) media. The inserts (A,B) show the ζ-potential ± SD of fullerenol in an aqueous solution**.** The measurements were carried out at 25 °C. The mean hydrodynamic radii and SD values of the analyzed particles were obtained by the DynaLS software (Vers. 2.9.1, Dr. Alexander Goldin, Alango Ltd., Tirat Carmel, Israel).
